# Impact of diode laser application alongside open flap debridement in periodontitis treatment

**DOI:** 10.6026/973206300213710

**Published:** 2025-10-31

**Authors:** Rupam Kumari Rupam, Kausar Parwez Khan, Shalini Sinha, Iswar Chandar Vidyasagar, Saurabh Kumar, Shimiyan Debbarma

**Affiliations:** 1 Department of Periodontology and Implantology, Mithila Minority Dental College and Hospital, Darbhanga, Bihar, India; 2 Department of Oral Medicine and Radiology, Agartala Government Dental College and IGM Hospital, Agartala, Tripura, India

**Keywords:** laser dentistry, flap surgery, periodontitis, wound healing, surgical periodontology

## Abstract

Periodontitis is a progressive inflammatory disease affecting the tooth-supporting structures. Although open flap debridement (OFD) is effective in managing advanced cases, complete elimination of pathogenic biofilms remains challenging. Diode lasers are popularly used as adjunctive aids owing to their bactericidal and stimulatory properties. Therefore, it is of interest to assess whether the efficacy of diode laser along with open flap debridement in improving clinical outcomes in severe periodontitis. The study involved 60 systemically healthy individuals with generalized periodontitis and probing pocket measuring more than 5 mm divided into test and control group. Clinical parameters-probing pocket depth (PPD), clinical attachment level (CAL), modified sulcular bleeding index (mSBI), wound healing index (WHI) and visual analog scale (VAS) for pain-were assessed at baseline, 3 months and 6 months. Group B demonstrated significantly better wound healing scores on Day 3 and Day 7 while significant improvements were observed in post-operative pain, CAL, PPD and mSBI at 6 months. Diode lasers offer a promising tool for optimizing the surgical management of severe periodontal cases.

## Background:

Periodontitis remains one of the predominant inflammatory oral diseases affecting the supporting structures of the teeth, identified with progressive gingival loss, bone resorption and periodontal pocket formation. It is primarily caused by the accumulation of pathogenic microbial biofilms and is influenced by immune defence of the host and factors affecting the environment. It may result in tooth mobility and eventual tooth loss, significantly affecting oral function and quality of life [1]. Non-surgical treatment modalities such as oral prophylaxis using mechanical scaling aim to remove calculus and plaque. However, in moderate to severe cases, where deeper periodontal pockets and complex root anatomies are present, surgical intervention such as open flap debridement (OFD) is often required for effective decontamination [2]. Open flap debridement surgery is a leading treatment for deep periodontal pockets when mechanical removal of calculus and plaque is insufficient. It involves lifting the gingival tissue to remove diseased tissue and thoroughly debride root surfaces. The flap is then closed, allowing for healing and potential regeneration of lost bone and periodontal ligament using grafts or growth factors, aiming to restore periodontal health and tooth support [3, 4]. Conventional mechanical debridement can temporarily reduce sub gingival levels of other periodontal pathogens. However, it often falls short in completely eliminating bacterial niches located in hard-to-reach areas such as deep periodontal pockets, root concavities and furcation sites. To overcome these limitations and promote more effective and minimally invasive periodontal healing, the use of lasers has been proposed as a promising adjunctive approach in periodontal therapy [5]. Laser surgeries are widely popular as they alter the subgingival microflora, offering a significant advantage over antibiotics by eliminating the risk of side effects and minimizing the potential for bacterial resistance [6]. The soft tissue lasers commonly used in dentistry are carbon dioxide, Nd:YAG, argon, Er:YAG, Er,Cr:YSGG and diode lasers-of which is particularly suited for procedures such as alteration of the gingiva, coagulation of oral mucosa, soft tissue curettage and sulcular debridement due to its minimal interaction with hard tissues [7]. Diode lasers ablate tissue mainly through thermal effects, creating a coagulation layer with minimal damage to underlying bone. They are ideal for soft tissue surgeries, periodontal therapy and peri-implantitis, with some hard tissue uses like root canal disinfection and tooth whitening. In addition to their compact size and cost-effectiveness, diode lasers offer several clinical advantages, including dry and bloodless surgical fields, instant sterilization, reduced tissue trauma and minimal postoperative pain and swelling. Several studies have highlighted the bactericidal effects of diode lasers on periodontopathogens, along with their ability to reduce inflammation and support soft tissue healing [8, 9]. However, clinical evidence regarding their added benefit when combined with traditional surgical techniques like OFD is still evolving, with limited data available on their long-term impact on clinical outcomes. Therefore, it is of interest to evaluate the effectiveness of diode laser as an adjunct to open flap debridement in the treatment of chronic periodontitis.

## Materials and Methods:

## Study design:

The current study was conducted in Department of Periodontology and Oral Implantology, Mithila Minority Dental College & Hospital, Darbhanga, Bihar. Ethical approval was obtained prior to starting the study (EC/New/Inst/2023/4152/Ref 06).

## Sample size estimation:

The sample size was calculated using G Power software (version 3.0.10) with an effect size of 0.250 [6], 5% precision, 95% confidence level and 80% power. A longitudinal study with minimum of 101 sites was calculated; rounded to 120 sites (60 patients per group).

## Eligibility criteria:

The study population consisted of systemically healthy individuals aged between 25 to 60 years, diagnosed with chronic periodontitis presenting with probing pocket depths (PPD) greater than 5 mm, for which open flap debridement was indicated. Participants who consented to the study were included. Patients undergoing antibiotic medication in the past six months, with pregnancy or currently lactating, current smokers, or with systemic conditions that could interfere with periodontal status or wound healing were excluded. Additionally, individuals with poor oral hygiene compliance or those deemed uncooperative with the study protocol were also excluded.

## Study procedure:

Following initial screening based on the inclusion and exclusion criteria, eligible participants were randomly divided into two groups. Group A received conventional therapy consisting of scaling and root planing (SRP) followed by open flap debridement (OFD), while Group B received SRP followed by OFD with adjunctive diode laser application. All participants initially underwent SRP as part of the non-surgical phase of therapy. After a healing period of four weeks, surgical procedures were performed under local anaesthesia. Full-thickness muco-periosteal flaps were elevated to expose the affected areas. Granulation tissue was thoroughly removed and the surgical sites were irrigated with sterile saline. In Group B, following debridement, a 980 nm diode laser (Woodpecker LX16 Plus Dental Diode Laser) was applied to the inner surface of the reflected flap using a 2-watt continuous wave mode. After laser application, the flaps in both groups were readjusted using 4-0 silk sutures. Postoperative care was kept similar for the test and control group to avoid bias. Patients were prescribed suitable medication when required and were instructed to use 0.12% chlorhexidine gluconate mouthwash twice daily for one week. Clinical parameters of probing pocket depth. Clinical attachment level modified sulcular bleeding index, wound healing index and visual analog scale scores for post-operative pain were assessed.

## Study tools:

The wound healing index [10] employs a 5-point scoring system, ranging from 1 (poorest healing) to 5 (excellent healing). It evaluates clinical parameters such as healing status, tissue color, bleeding on palpation, presence of granulation tissue and suppuration. The Modified Sulcular Bleeding Index is a clinical indicator of gingival inflammation, particularly bleeding on gentle probing, which reflects the severity of inflammation and vascular response in the gingival tissues [11]. The Visual Analog Scale is a subjective measure of pain perception, commonly used to evaluate postoperative discomfort [12]. These parameters were assessed at baseline and at designated follow-up intervals to compare the therapeutic outcomes between the two groups.

## Statistical analysis:

The collected data was computed in Microsoft excel sheet and Independent t test was applied. A p value of <0.05 was considered to be statistically significant.

## Results:

Table 1 (see PDF) shows the comparison of the wound healing between the groups at Day 3 and Day 7. Group B demonstrated significantly better wound healing scores (1.70 ± 0.59) compared to Group A (3.06 ± 0.82), with a p value (p = 0.00). By day 7, both groups showed improvement, but Group B maintained superior healing (mean 4.70 ± 0.46) compared to Group A (4.13 ± 0.68) and it remained significant (p = 0.00). Pain perception was notably lower in the Group B, as shown in Table 2 (see PDF). On day 3, Group B reported a mean pain score of 2.10 ± 0.96, whereas Group A had a higher mean score of 6.70 ± 0.98 (p = 0.00). By day 7, the mean pain score reduced in both groups; however, Group B continued to show significantly less pain (0.33 ± 0.47) compared to Group A (3.53 ± 0.68), with the difference remaining highly significant (p = 0.00). Table 3 (see PDF) compares the clinical attachment loss between the two study groups. At baseline, the difference between groups was not statistically significant (p = 0.071). However, after 3 months, Group B showed significantly greater gain in attachment (mean CAL: 3.70 ± 0.63) compared to Group A (5.80 ± 0.85) (p < 0.001). This improvement was further pronounced at 6 months, where Group B recorded a mean CAL of 1.13 ± 0.44, significantly better than Group A's mean of 4.93 ± 0.66 (p < 0.001). Table 4 (see PDF) compares the periodontal pocket depth (PPD) between Group A and Group B. There was no significant difference in baseline PPD between the two groups (p = 0.93). At 3 months, Group B demonstrated a greater reduction in pocket depth (4.70 ± 0.53) compared to Group A (5.76 ± 0.47) (p = 0.00). This trend continued at 6 months, with Group B showing significantly lower pocket depth (4.11 ± 0.69) compared to Group A (5.27 ± 0.40) (p = 0.00). At baseline, no significant difference in sulcular bleeding (Table 5 - see PDF) was observed between the groups (p = 0.56). However, after 3 months, a significant reduction was observed in Group B (1.13 ± 0.28) as compared to Group A (2.36 ± 0.26) (p < 0.001). At 6 months, Group B continued to demonstrate a lower bleeding index (0.86 ± 0.40) versus Group A (1.01 ± 0.17), with the difference remaining statistically significant (p = 0.04).

## Discussion:

The currently conducted study was conducted to assess the effectiveness of diode laser as an aide to OFD for the management of chronic periodontitis. The findings of the current study demonstrated that the use of diode laser as an aide significantly improved clinical parameters as opposed to conventional periodontal therapies. Parameters such as PPD, CAL, mSBI, WHI and VAS scores for pain showed statistically significant improvements in the laser group, at different time intervals, post operatively. The significant reduction in probing pocket depth and gain in clinical attachment loss observed in Group B (laser-assisted OFD) can be attributed to the bactericidal and de-epithelializing effects of the diode laser [13]. Diode lasers are known to facilitate better debridement of inflamed and granulation tissues and exert deep disinfection of the periodontal pocket, particularly in areas inaccessible to mechanical instrumentation. This may have contributed to enhanced soft tissue healing and reattachment, as evident from the superior CAL gain observed in Group B [14, 15]. The findings of the study are consistent with prior studies which reported improved periodontal parameters when lasers were used adjunctively with mechanical debridement [7, 16]. Wound healing was also significantly better in the Group B on both day 3 and day 7 postoperatively. This may be explained by the bio-modulatory effects of laser irradiation, which has been shown to enhance fibroblast proliferation, collagen synthesis and angiogenesis. These properties likely promoted faster tissue repair and maturation in the early healing phase. The laser has potential to help in lower post-operative pain as it helps in sealing nerve endings and lymphatic vessels thus reducing inflammation and stimulation of the neural network [6, 17]. The reduction in bleeding scores (mSBI) further supports the anti-inflammatory effects of diode laser therapy. As laser application coagulates the vascular plexus and reduces bacterial load, it may have effectively modulated the inflammatory response in the periodontal tissues, resulting in less bleeding upon probing [18, 19].

## Strengths and limitations:

The study's strengths include the inclusion of multiple clinical parameters across multiple follow-up intervals provides a comprehensive assessment of healing and treatment efficacy. Furthermore, the laser settings and procedural protocols were standardized, reducing the likelihood of operator-induced variability. However, certain limitations must be acknowledged. First, this study relied exclusively on clinical parameters and did not assess microbiological or radiographic outcomes, which could have provided a deeper understanding of underlying biological effects. Secondarily, the follow up period of six months do not necessarily prove the long term stability of improvements in patient outcomes. Moreover, while patient compliance was monitored, behavioral and oral hygiene practices may have influenced healing outcomes. Lastly, the sample size being small the findings cannot be generalized to the population. Further studies with larger sample sizes, longer follow-up durations and inclusion of microbiological, radiological and histological data are necessary to substantiate these findings and to refine laser protocols for broader clinical application.

## Conclusion:

We show that the aided use of a diode laser with open flap debridement significantly enhances clinical outcomes and improves healing through its bactericidal and anti-inflammatory effects.
